# Canopy distribution and microclimate preferences of sterile and wild Queensland fruit flies

**DOI:** 10.1038/s41598-021-92218-8

**Published:** 2021-06-21

**Authors:** Jess R. Inskeep, Andrew P. Allen, Phillip W. Taylor, Polychronis Rempoulakis, Christopher W. Weldon

**Affiliations:** 1 Applied BioSciences, Macquarie University, North Ryde, NSW 2109 Australia; 2 Department of Biological Sciences, Macquarie University, North Ryde, NSW 2109 Australia; 3 New South Wales Department of Primary Industries, Ourimbah, NSW 2258 Australia; 4 Department of Zoology and Entomology, University of Pretoria, Private Bag X20, Hatfield, 0083 South Africa; 5 Vector Control, Hawaii Department of Health, Kahului, HI 96732 USA

**Keywords:** Animal behaviour, Entomology, Behavioural ecology, Invasive species

## Abstract

Insects tend to live within well-defined habitats, and at smaller scales can have distinct microhabitat preferences. These preferences are important, but often overlooked, in applications of the sterile insect technique. Different microhabitat preferences of sterile and wild insects may reflect differences in environmental tolerance and may lead to spatial separation in the field, both of which may reduce the control program efficiency. In this study, we compared the diurnal microhabitat distributions of mass-reared (fertile and sterile) and wild Queensland fruit flies, *Bactrocera tryoni* (Froggatt) (Diptera: Tephritidae). Flies were individually tagged and released into field cages containing citrus trees. We recorded their locations in the canopies (height from ground, distance from canopy center), behavior (resting, grooming, walking, feeding), and the abiotic conditions on occupied leaves (temperature, humidity, light intensity) throughout the day. Flies from all groups moved lower in the canopy when temperature and light intensity were high, and humidity was low; lower canopy regions provided shelter from these conditions. Fertile and sterile mass-reared flies of both sexes were generally lower in the canopies than wild flies. Flies generally fed from the top sides of leaves that were lower in the canopy, suggesting food sources in these locations. Our observations suggest that mass-reared and wild *B. tryoni* occupy different locations in tree canopies, which could indicate different tolerances to environmental extremes and may result in spatial separation of sterile and wild flies when assessed at a landscape scale.

## Introduction

In nature, abiotic conditions fluctuate significantly through the day, and seasonally through the year. These fluctuations often present unfavorably high and/or low temperatures to animals, and may reduce their abilities to survive and reproduce^1,2^. Insects in particular, due to their small size, adopt a range of strategies to tolerate and limit water loss^3^. For example, insects often rely on shelter-seeking behaviors to avoid prolonged exposure to harmful conditions^4–6^. For insects inhabiting plant foliage, arboreal canopies are complex three-dimensional structures that affect physical environmental variables such as shading from solar radiation and leaf temperature while also being modified by biological processes^7,8^. Herbivorous insects may affect leaf microclimates through their feeding^9,10^ and shape canopy structures^11^ to improve their surrounding conditions. Cell, phloem and xylem feeders are in direct contact with a source of water that can improve their tolerance of thermal extremes^12,13^. Even among insects not feeding on plant tissues, stomatal opening, which is influenced by season, regional climate, local topography, canopy and plant structure, increases transpiration and acts to reduce leaf temperature though evaporative cooling^9^.

The microhabitats occupied by insects are an important consideration for pest management strategies that rely on releases of live insects^14^. The sterile insect technique (SIT) is one such strategy, which involves the mass rearing, reproductive sterilization, and release of large numbers of adult insects into nature^15^. Mating between sterile males and wild females results in sterile egg production, and therefore a generational decline in the wild population due to infertility. This pest management strategy is used frequently, and increasingly, to manage pestiferous fruit fly (Diptera: Tephritidae) populations across the world^16–18^. Released sterile flies must survive, disperse, and mature, and then encounter and mate with wild flies to reduce fertility in the wild population^15,19,20^. While a suite of standardized quality control assays are routinely performed in fruit fly SIT programs to monitor production standards, and numerous studies have identified factors influencing sexual competence^21–26^, less is known about the ecological competence of sterile fruit flies or about how the behavior and microhabitat preferences of sterile flies align with that of their wild counterparts in the field.

Sterile fruit flies have sometimes been found to be less adept at dispersing over large distances compared to wild conspecifics^27^, and their distribution in the field may be particularly affected by water and nutrient availability^28,29^. At a finer scale, though, the locations of sterile and wild fruit flies within tree canopies have seldomly been considered. Through the day, flies forage for nutrition while also avoiding threats to their survival, such as from predators and hazardous abiotic conditions. *Bactrocera cacuminata*^30^ and *Ceratitis capitata*^31,32^ prefer dense foliage in the middle of the day, possibly to avoid high temperatures on sun-exposed leaves. *Anastrepha obliqua*^33^ and *A. striata*^34^ are less active during the middle of the day when temperatures are highest and feed (from leaf surfaces) more frequently in the morning and late afternoon when temperatures are cooler. However, there is a paucity of comparisons between sterile and wild flies with regard to their diurnal behaviors^35^, tree-canopy distributions and microhabitat preferences.

The present study considers the microhabitat locations and preferences of the Queensland fruit fly, *Bactrocera tryoni* (Froggatt) (Diptera: Tephritidae). This pest fruit fly is native to eastern Australia but has invaded southern and western regions of Australia^36^, New Zealand^37,38^, the United States^39^, and numerous islands in the Pacific^40^. *Bactrocera tryoni* is a formidable agricultural pest owing to its wide host range, relatively high mobility, rapid population growth, and long lifespan (reviewed by Clarke et al.^41^, Dominiak^42^). In regions where *B. tryoni* occurs, most commercially grown fruits are threatened^43^. Consequently, *B. tryoni* is recognized globally as a high-level quarantine pest and regions with these flies are subject to trade restrictions and strict phytosanitary requirements^38,44–46^. In an effort to manage pest populations of *B. tryoni*, and prevent it from establishing a more extensive south-eastern range, applications of the sterile insect technique (SIT) are being increasingly relied upon in Australia^47^. Laboratory studies have suggested that domesticated or mass-reared *B. tryoni,* such as are used in SIT programs, differ from wild flies in their environmental preferences^48^ and tolerances^49^. However, the impacts of such behavioral and physiological differences on distribution of *B. tryoni* in the field has not been studied.

We here assess effects of mass rearing and irradiation on daytime canopy distributions, behaviors, and microclimate preferences of *B. tryoni* in field cages, comparing (1) fertile mass-reared, (2) sterile mass-reared, and (3) wild flies. We expected all flies to avoid microhabitats with adverse abiotic conditions such as high temperature and light intensity, and low humidity. Amongst the tested groups, we predicted a greater degree of avoidance of unfavorable microhabitat conditions in mass-reared *versus* wild *B. tryoni*, due to the mild and stable abiotic conditions that mass-reared flies are produced under and appear to be adapted to^48,49^. Finally, we included measurements of the spatial locations (height from ground, distance from canopy center, and upper or lower leaf surface) of daytime behaviors.

## Materials and methods

### Fly types

Mass-reared flies were sourced from cultures maintained at two locations – the Elizabeth Macarthur Agricultural Institute and Macquarie University – that were held in 40 × 40 × 110 cm wire mesh cages containing ca. 7,500 flies and fed larval diets based on lucerne chaff^50^ and agar gel^51^, respectively. Both fertile and sterile mass-reared fly types were from both locations. Sterilization was induced by exposing mass-reared pupae to 70 Gy of gamma radiation, under hypoxia, using cobalt-60 sources located at either the Australian Nuclear Science and Technology Organization or Macquarie University. Wild flies were sourced from loquats (*Eriobotrya japonica* [Thunb.] Lindl.) collected from trees in Auburn, NSW, Australia. Climactic conditions at the field site are provided in Supplementary Materials (Table S1). Loquats collected in the field were naturally infested with wild *B. tryoni* eggs and/or larvae. Containers of artificial diet (for fertile and sterile mass-reared flies) and infested fruits (for wild flies) were placed in wooden trays with plastic mesh bases that were suspended over a 20 mm deep bed of vermiculite (Grade 1, Ausperl, Orica Australia Pty. Ltd., Banksmeadow, NSW, Australia) in plastic bins. The vermiculite was sieved gently at weekly intervals to collect pupae. All life stages of flies from mass-reared and wild (including loquat fruits which contained wild eggs and larvae) cultures were held at 25 ± 0.5° C, 65 ± 5% RH, and with a 1:11:1:11 dawn:day:dusk:night photoperiod.

From eclosion, all flies were provided unrestricted access to white granulated sugar (CSR White Sugar) and yeast hydrolysate (MP Biomedicals #02,103,304) (3:1 vol:vol) as food, and sponges soaked in water. Flies were sexed and separated 2–4 days after eclosion, before they reached sexual maturity^52,53^. Adults were held under the same conditions as pupae prior to release into field cages (see below).

### Fly marking and release

Following a tagging method similar to McInnis et al.^54^, 50 male and 50 female adults of each fly type (fertile mass-reared, sterile mass-reared, and wild) were individually marked with labels of colored paper (1.5 mm × 1.5 mm, 3 pt Arial font) when 2–4-days old. Label colors denoted fly type and label numbers (1–50 for each fly type and sex) were unique for each individual. Label colors were rotated between the fly types for each replicate. To attach the label, each fly was first immobilized by placing it into a vial and chilling the vial on crushed ice for 60 s. Then, a small drop of white non-toxic enamel paint (Tamiya Inc., Shizuoka, Japan) was applied to the middle of the thorax using a bristle from a broom. Finally, the label was carefully pressed onto the paint using a pair of fine forceps. After applying labels, flies quickly resumed normal activity after exposure to ambient temperatures.

### Field cages

When adults were 6 days old, the tagged 50 males and 50 females of each of the three fly types (300 flies in total) were released into field cages (10 replicates). Field cages (3 m diameter × 2.2 m high cylindrical-shaped [Synthetic Industries, Dalton, GA, USA]) were large enough for a single observer to walk inside and record behaviors. In the center of each field cage, three potted plants (*Citrus aurantium* L. and/or *C. meyeri* Yu. Tanaka) were arranged to create a single continuous canopy. Two food stations (10 g of a 3:1 [vol:vol] mixture of white granulated sugar and yeast hydrolysate placed on a 9 cm plastic Petri dish) and two water stations (1 L plastic container containing water and an exposed sponge) were suspended in the middle of each canopy. Measurements of flies observed at food or water stations were not taken, but all three fly types were observed utilizing the stations. A vertical wooden pole was erected in the center of the canopy from which measurements of fly distance from canopy center were taken (see below). Field cages were placed in full sun under a transparent 6 × 6 m square-shaped clear polycarbonate roof for rain protection.

### Data collection

Observations on spatial distributions, abiotic conditions, and behaviors (described below) were carried out 9:00–10:00 (‘morning’), 11:30–12:30 (‘midday’), 14:00–15:00 (‘early afternoon’), and 16:30–17:30 (‘late afternoon’). Observations were carried out when flies were 8, 12, 16, and 20 days old. Fly identity (based on sex, label color and label number) was recorded for each observation. We also recorded the behavior and location of individual flies, and the abiotic conditions of the leaf surfaces that they occupied (see below). After recording data for one sampled fly, a single observer moved in a circle around the tree and recorded data for the next fly to be seen. When recording flies, the observer alternated between scanning from the canopy center toward the periphery and vice versa. Also, the observer alternated between scanning from the canopy top to bottom and vice versa. All flies encountered by the observers were recorded, but the tree canopies were large enough that sometimes several steps around the tree were taken before a new fly was found to measure. From all data recorded (see below), observations of each fly took on average 9.0 ± 0.28 (SE) min. Observations were carried out mid- to late-summer; April 28—May 10 in 2008, February 28—March 14 in 2009, and January 9—April 10 in 2017.

Observations of behaviors were recorded in detail, but only four behaviors were common, (1) resting, (2) grooming, (3) walking, and (4) feeding. Flies were considered ‘resting’ when they showed no signs of movement, and their mouthparts were not pressed against a surface. They were considered ‘grooming’ when they rubbed their legs together or moved their two forelegs over their eyes, bodies, or wings. They were considered ‘feeding’ when they were stationary with mouthparts pressed against a surface or when taking a few steps before pausing for any duration of time to again press their mouthparts to a surface. Finally, they were considered ‘walking’ when they were moving on or between leaf surfaces while exhibiting no indications of feeding.

Spatial locations of flies were recorded by measuring the distance from the fly to the ground (‘height’), and the distance from the fly to the pole marking the center of the canopy (‘radius’), using a laser distance meter (Zamo, Bosch, Stuttgart, Germany). The leaf surface (upper [adaxial] or lower [abaxial]) on which the fly was located was also recorded.

Abiotic conditions (temperature, humidity, and light intensity) were measured above and below the leaf on which the fly was observed. Conditions were measured using the HM40 handheld temperature and humidity probe (Vaisala, Helsinki, Finland), which had a precision of ± 0.2 °C and ± 1.5% RH. The probe was placed ~ 1 cm above or below the leaf surface, which represented the air volume in which flies were located when perched on the leaf (= leaf boundary). Light intensity was measured using a handheld light intensity meter (LX-107HA, Lutron, Taipei, Taiwan) that was placed 2 cm above or below the measured leaf surfaces.

### Model selection and data analysis

Correlates of the spatial locations of flies, as indexed by height and radius, were assessed using R statistical software^55^ by fitting linear mixed-effects models using version 1.1–21 of the R package lme4^56^. When multiple measurements of the same fly (identified by unique combinations of sex, label color and label number) were present in the data, one measurement was randomly selected and kept, and the other measurements removed, to avoid pseudoreplication. We adopted this approach rather than incorporating fly ID as an additional random effect because the vast majority of flies were measured only once. Consequently, only 29.8% of all observations were removed using this procedure, leaving 1,001 observations for analysis. Preliminary analyses showed that flies used in replicates in 2008, 2009, and 2017 did not differ in their spatial locations (Supplementary Materials, Table S2 & Figures S1–S2), so replicate and year were excluded from the final models (see below).

The two response variables, height and radius, were analyzed separately considering the same two sets of candidate predictor variables. First, to assess diurnal trends in the spatial distributions of flies, and the extent to which these trends differed with fly attributes, we fit a series of models that included one or more of the following variables (and their two-way interactions) as candidate fixed factors: ‘time of day’, ‘fly type’, ‘sex’, and ‘age’. Second, to assess the extent to which diurnal trends were driven by proximate environmental factors, we fit a different set of models that included one or more of the following variables (and their two-way interactions) as fixed factors: ‘temperature’, ‘relative humidity’, ‘light intensity’, ‘fly type’, ‘sex’, and ‘age’. Measurements of ‘temperature’, ‘relative humidity’, and ‘light intensity’ used in this analysis were from the air boundaries (~ 1 cm) above or below leaf surfaces where measured flies had alighted (e.g., abiotic conditions of the lower leaf boundary were used if the fly was on the lower leaf surface during observation). The variable ‘day’ was included as a random factor in all of the fitted models to account for potential effects of other unmeasured variables that varied from day to day. For each set of candidate predictors, all model permutations were fitted using the ‘dredge’ function in the MuMIn package^57^. Fitted models were then compared using the corrected Akaike Information Criterion (AICc)^58^, which balances model fit and complexity based on information theoretic arguments. The model with the lowest AICc score, AICc_min_, is judged to be ‘best’ according to this criterion, and models within 4 AICc units of this best model are often judged to have substantial support^59^. To aid in model selection, a weight was calculated for each model *i* as

$$w_{i} = {\text{exp}}\left( {\left( {{\text{AICc}}_{{{\text{min}}}} - {\text{AICc}}_{i} } \right)/2} \right)/\mathop \sum \limits_{{i = 1}}^{n} {\text{exp}}\left( {\left( {{\text{AICc}}_{{{\text{min}}}} - {\text{AICc}}_{i} } \right)/2} \right)$$

based on the *n* models within 4 AICc units of ${\text{AICc}}_{{{\text{min}}}}$. Then, to assess the relative contribution of each variable to model parsimony, a variable weight was calculated as the sum of the weights for all the models that incorporated that variable^59^. This AICc sum helps determine a variable’s contribution to model parsimony^60^, but not its effect size. Categorical variables judged to be significant (α < 0.05) based on calculated Type III F statistics were subjected to Tukey post-hoc tests using the package emmeans^61^.

We also investigated how the average heights and radii of flies varied in relation to the fixed factors ‘behavior’, ‘fly type’, and their interaction, using a linear mixed model and treating ‘day’ as a random factor. Categorical variables judged to be significant (α < 0.05) based on calculated Type III F statistics were subjected to Tukey post hoc tests using the package emmeans^61^.

To assess differences in abiotic conditions by leaf surface (i.e., upper or lower), three linear model ANOVAs were performed with temperature, relative humidity, and light intensity as the response variables and leaf surface, time of day (i.e., morning, midday, early afternoon, and late afternoon) and their interactions as fixed factors. To assess the occurrence of behaviors by leaf surface (i.e., upper or lower), we compared the factors ‘behavior’ and ‘fly type’ by leaf surface using binomial tests in the base package of R^55^. *P* values in binomial tests were calculated based on the null hypothesis of behaviors occurring on either the upper or lower surfaces of leaves with equal probability (*P* = 0.5). Descriptive statistics in the texts of the Result section are given as mean values ± SE.

### Ethical approval

All applicable international, national, and/or institutional guidelines for the care and use of animals were followed.

## Results

### Model selection

For the height data, the best model (AICc_min_) that included abiotic variables (temperature, humidity, and light intensity) was 52.4 AICc units lower than the best model that included ‘time of day’ (levels: morning, midday, early afternoon, late afternoon) (Supplementary Materials, Tables S3, S4). Thus, including abiotic variables substantially improved predictive power. Given that the weights of all three of these abiotic variables, along with two interactions, were all equal to 1, with highly significant *P* values for the AICc_min_ model, all three abiotic variables appeared to be important drivers of height (Table 1). For the radius data, the AICc scores were also generally lower when abiotic variables were included rather than ‘time of day’ (4.3 AICc unit difference for the two best models, Supplementary Materials, Tables S5, S6), but their effects on radius were relatively weak, as compared to height, judging by the lower weights of the abiotic variables in the AICc_min_ model (Table 2). Therefore, the final models for height and radius included abiotic conditions but not time of day (Tables 1, 2). However, descriptive and graphical results of time of day are provided as an alternative interpretation (Figs. 3, 4).

**Table 1:** Mixed-model results for the best model (AICc_min_; bolded) with height (mm) as response variable.

Factor Type	Factor/Level		AICcW	N models	Estimate	SE	Statistic	DF	*P* value
Fixed		Intercept	–	–	3317.27	412.85	–	–	–
	**Fly type**		**1.00**	**74**			**19.78**	**2, 943.30**	**2.4 × 10**^**–10**^
		Wild (reference)			**–**	**–**			
		Fertile			**− 122.74**	**19.71**			
		Sterile			**− 105.33**	**21.06**			
	**Sex (Female)**		**0.90**	**66**	**31.61**	**16.65**	**2.60**	**1, 938.70**	**0.06**
	Age (Days)		0.02	3	**–**	**–**	**–**	**–**	**–**
	**Temperature (°C)**		**1.00**	**74**	**− 79.98**	**17.37**	**21.54**	**1, 923.70**	**4.7 × 10**^**–6**^
	**Humidity (%)**		**1.00**	**74**	**− 14.64**	**3.91**	**11.32**	**1, 858.71**	**1.9 × 10**^**–4**^
	**Light (Log**_**10**_** lux)**		**1.00**	**74**	**− 90.69**	**28.73**	**12.43**	**1, 905.91**	**1.6 × 10**^**–3**^
	Fly type:Sex		0.08	9	**–**	**–**	**–**	**–**	**–**
	Fly type:Age		**–**	0	**–**	**–**	**–**	**–**	**–**
	Fly type:Temperature		0.15	14	**–**	**–**	**–**	**–**	**–**
	Fly type:Humidity		0.23	19	**–**	**–**	**–**	**–**	**–**
	Fly type:Light		0.24	21	**–**	**–**	**–**	**–**	**–**
	Sex:Age		**–**	0	**–**	**–**	**–**	**–**	**–**
	Sex:Temperature		0.31	25	**–**	**–**	**–**	**–**	**–**
	Sex:Humidity		0.25	21	**–**	**–**	**–**	**–**	**–**
	Sex:Light		0.19	19	**–**	**–**	**–**	**–**	**–**
	Age:Temperature		**–**	0	**–**	**–**	**–**	**–**	**–**
	Age:Humidity		**–**	0	**–**	**–**	**–**	**–**	**–**
	Age:Light		**–**	0	**–**	**–**	**–**	**–**	**–**
	**Temperature:Humidity**		**1.00**	**74**	**0.77**	**0.16**	**20.28**	**1, 760.26**	**1.8 × 10**^**–6**^
	**Temperature:Light**		**1.00**	**74**	**3.84**	**1.32**	**11.63**	**1, 927.30**	**3.7 × 10**^**–3**^
	Humidity:Light		0.45	33	**–**	**–**	**–**	**–**	**–**
Random	**Day (Variance)**		**–**	**–**	**7318**	**85.55**	**44.04**	**1**	**3.2 × 10**^**–11**^

The sum of weights for the corrected Akaike Information Criterion (AICcW) and N models (total = 74) with AICcΔ < 4 for each factor are summarized here. Intercept has reference level Wild Male. The significance of fixed factors was assessed using F-statistic in a type III ANOVA with Satterthwaite’s method. The random factor was assessed using log-likelihood ratio with significance analyzed using a chi-square distribution.

**Table 2:** Mixed-model ANOVA results for the best model (AICc_min_; bolded) with radius (mm) as response variable.

Factor Type	Factor/Level		AICcW	N models	Estimate	SE	Statistic	DF	*P* value
Fixed		Intercept	**–**	**–**	458.31	36.27	**–**	**–**	**–**
	Fly type		0.05	1	**–**	**–**	**–**	**–**	**–**
	Sex (Male/Female)		0.22	4	**–**	**–**	**–**	**–**	**–**
	Age (Days)		**–**	0	**–**	**–**	**–**	**–**	**–**
	Temperature (°C)		0.40	5	**–**	**–**	**–**	**–**	**–**
	Humidity (%)		0.28	5	**–**	**–**	**–**	**–**	**–**
	**Light (Log**_**10**_** lux)**		**1.00**	**12**	**12.75**	**4.33**	**8.68**	**1, 878.6**	**3.3 × 10**^**–3**^
	Fly type:Sex		**–**	0	**–**	**–**	**–**	**–**	**–**
	Fly type:Age		**–**	0	**–**	**–**	**–**	**–**	**–**
	Fly type:Temperature		**–**	0	**–**	**–**	**–**	**–**	**–**
	Fly type:Humidity		**–**	0	**–**	**–**	**–**	**–**	**–**
	Fly type:Light		**–**	0	**–**	**–**	**–**	**–**	**–**
	Sex:Age		**–**	0	**–**	**–**	**–**	**–**	**–**
	Sex:Temperature		**–**	0	**–**	**–**	**–**	**–**	**–**
	Sex:Humidity		**–**	0	**–**	**–**	**–**	**–**	**–**
	Sex:Light		0.04	1	**–**	**–**	**–**	**–**	**–**
	Age:Temperature		**–**	0	**–**	**–**	**–**	**–**	**–**
	Age:Humidity		**–**	0	**–**	**–**	**–**	**–**	**–**
	Age:Light		**–**	0	**–**	**–**	**–**	**–**	**–**
	Temperature:Humidity		**–**	0	**–**	**–**	**–**	**–**	**–**
	Temperature:Light		0.13	2	**–**	**–**	**–**	**–**	**–**
	Humidity:Light		0.04	1	**–**	**–**	**–**	**–**	**–**
Random	**Day (Variance)**		**–**	**–**	**3366**	**58.02**	**43.66**	**1**	**3.9 × 10**^**–11**^

The sum of weights for the corrected Akaike Information Criterion (AICcW) and N models (total = 12) with AICcΔ < 4 for each factor are summarized here. The significance of fixed factors was assessed using F-statistic in a type III ANOVA with Satterthwaite’s method. The random factor was assessed using log-likelihood ratio with significance analyzed using a chi-square distribution.

Residuals of the height model were assumed to be normally distributed for fitting despite appearing slightly left-skewed (Supplementary Materials, Figure S3). This was due to a slight left-skew in the distribution of wild fly residuals (Supplementary Materials, Figure S4), which in turn was likely due to wild flies frequently alighting on the highest leaves in the canopies with no higher locations available. A Levene’s test for homogeneity of variance found no significant differences in the distributions of residuals between fly types in the height model (F_2,956_ = 2.40, p = 0.091). Radius model residuals appeared normally distributed (Supplementary Materials, Figure S5).

### Canopy distribution by fly type

**Figure 1:**
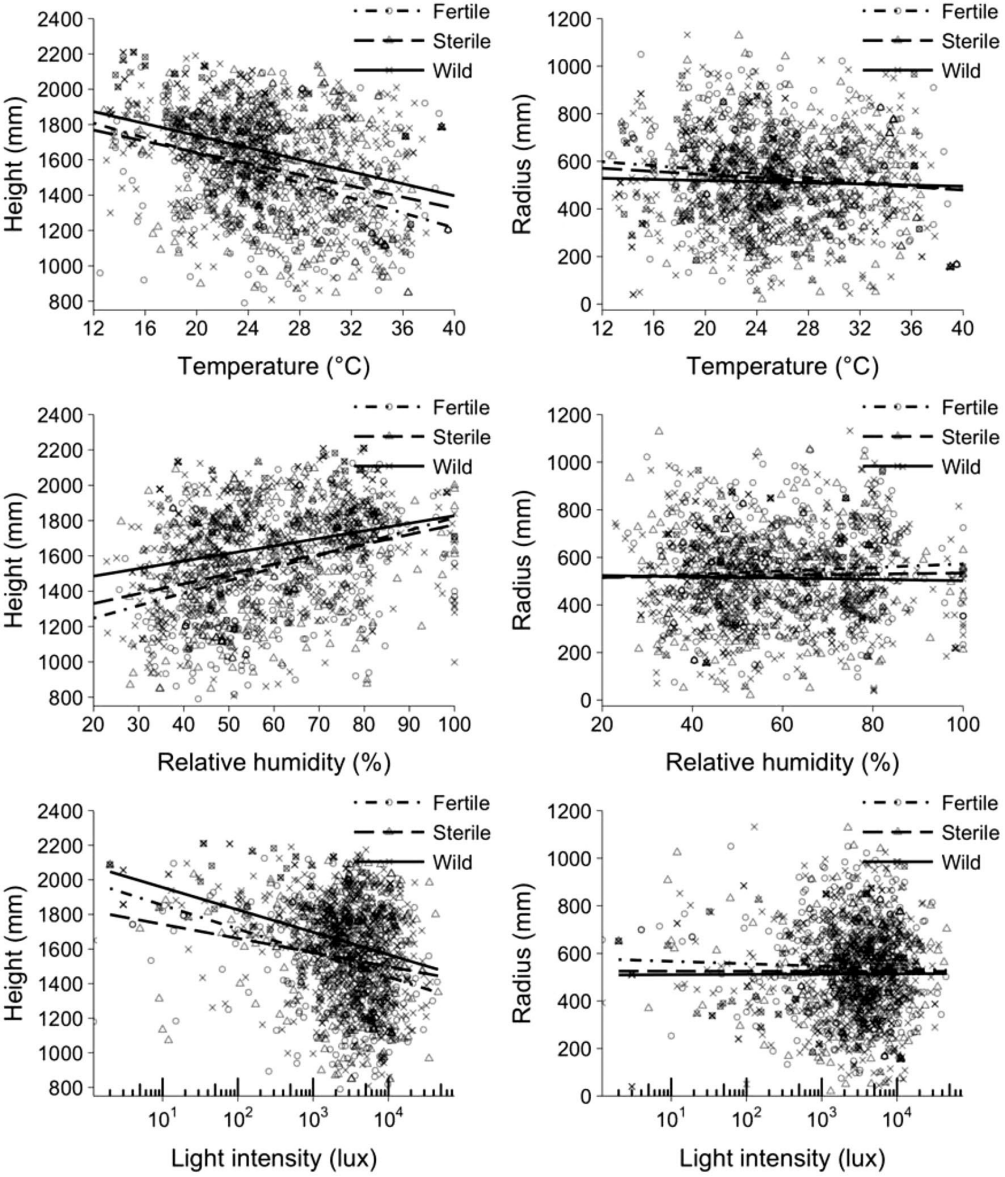
Distance from the ground (height) and distance from the canopy center (radius) of fertile mass-reared, sterile mass-reared and wild flies observed in *Citrus* tree canopies. Temperature, relative humidity, and light intensity were recorded from leaf surfaces where flies were located. Fitted lines represent mean heights or radii of the three fly types.

Fly types differed significantly by height (*P* < 0.001) (Table 1 & Fig. 1). Wild flies were higher in the canopies than fertile and sterile flies (both *P* < 0.001), respectively. Fertile and sterile flies exhibited no difference in height (*P* = 0.705). Females and males did not differ by height (*P* = 0.059). Fly type was not a significant factor affecting distance from canopy center (excluded from final model) (Table 2 & Fig. 1).

### Canopy distribution by abiotic condition

**Figure 2:**
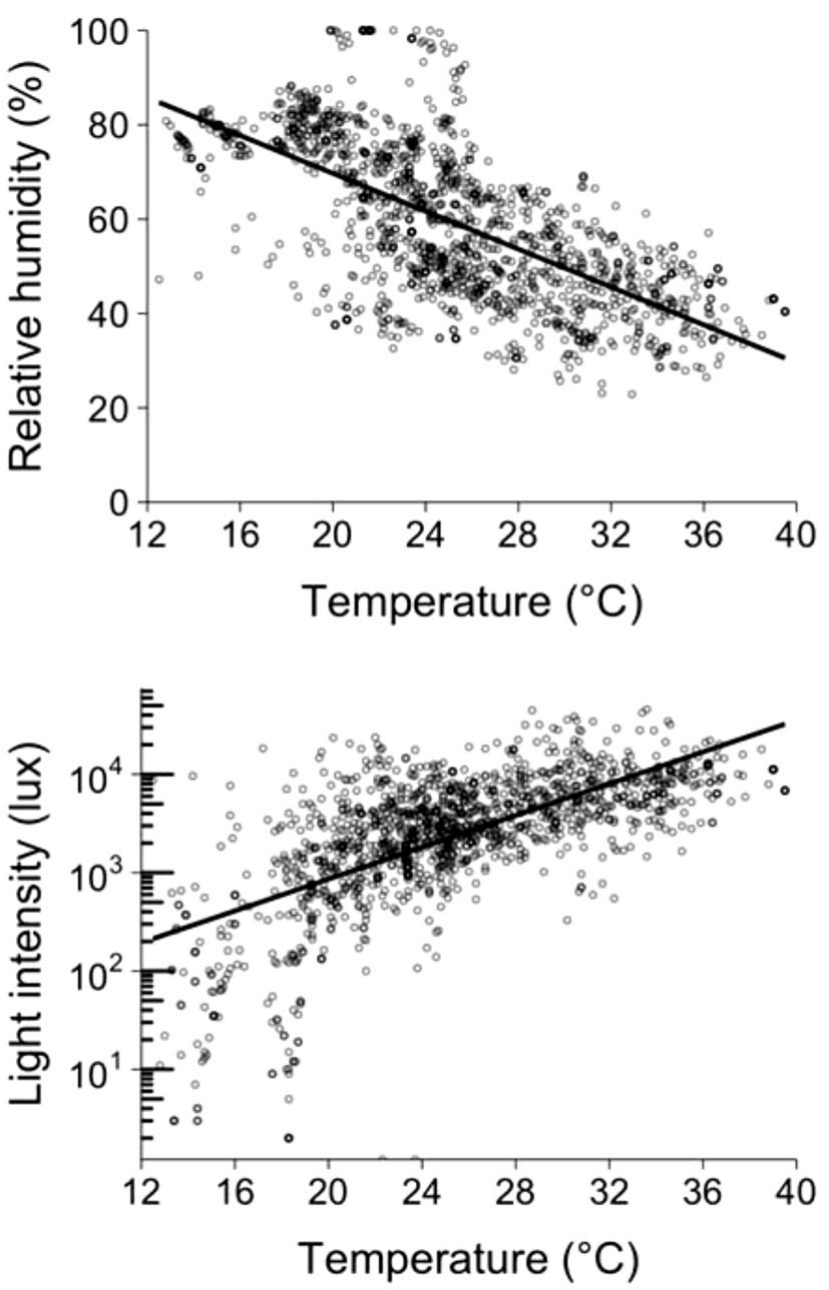
The relationships of relative humidity and light intensity to temperature for randomly measured leaves throughout the canopy.

Lower canopy locations had lower temperature (*P* < 0.001), lower light intensity (*P* < 0.001), and higher humidity (*P* < 0.001) (Table 1 & Fig. 1). Compared with leaves further from the canopy center, leaves closer to the canopy center had lower light intensity (*P* = 0.003), but did not differ in temperature or humidity (both excluded from the final model) (Table 2 & Fig. 1). Temperature was correlated negatively with relative humidity and positively with light intensity (Tables 1 & Fig. 2).

### Canopy distribution by behavior

**Figure 3:**
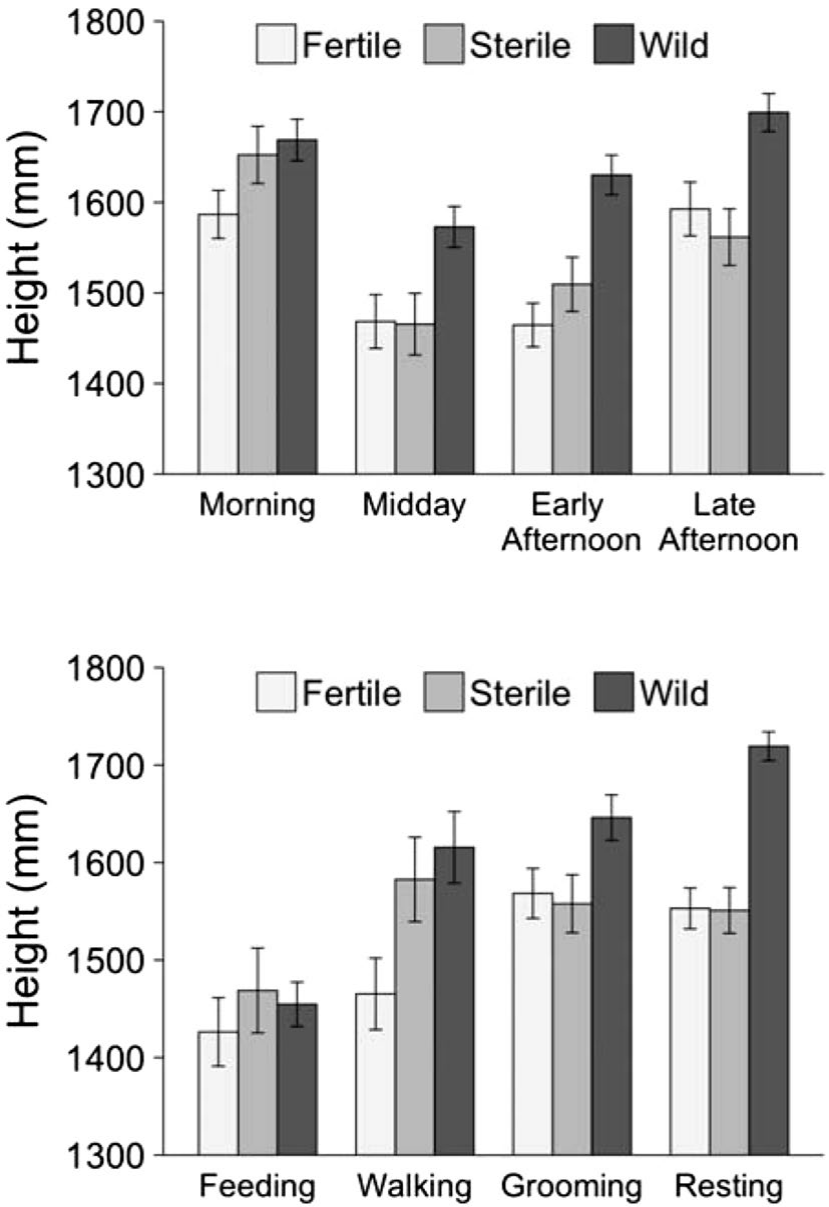
Average (± SE) height (mm) of flies in *Citrus* tree canopies by time of day and behavior.

**Figure 4:**
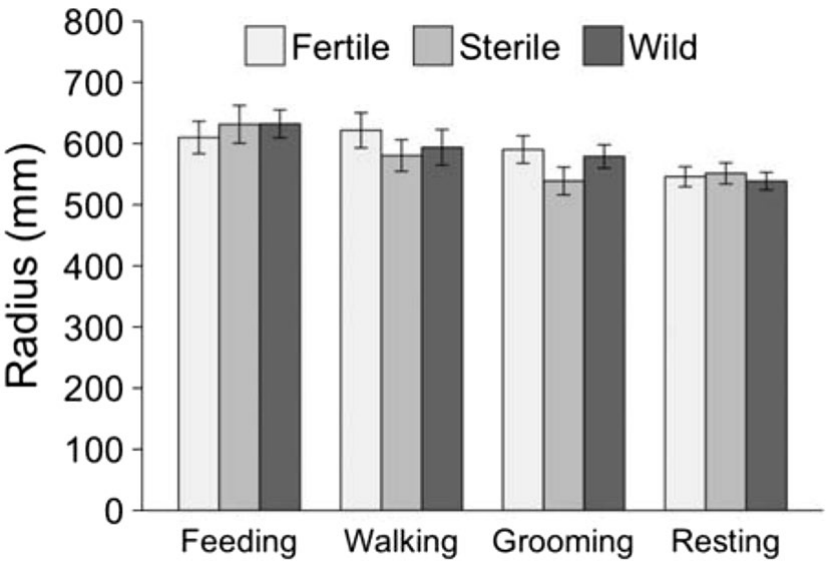
Average (± SE) radius (mm) of flies in *Citrus* tree canopies by behavior.

Behaviors differed significantly by height and radius (Table 3). Across all fly treatments, feeding occurred lower in the canopy than resting (*P* < 0.001), grooming (*P* < 0.001), and walking (*P* = 0.011), while resting, grooming, and walking occurred at the same height (all p > 0.05) (Fig. 3). There was a suggestive interaction between fly type and behavior (*P* = 0.052). This was because wild flies were resting higher than fertile (*P* < 0.001) and sterile flies (*P* < 0.001), but resting height between fertile and sterile flies did not differ (*P* = 0.712). Also, wild flies were walking at higher heights than fertile flies (*P* = 0.012), but there was no difference between fertile and sterile flies or between wild and sterile flies (both p > 0.05). For feeding and grooming, there was no difference in height by fly type (all p > 0.05). Behavior differed significantly by radius (*P* = 0.003) (Fig. 4). This was because, for all fly types, resting was observed closer to the canopy center than feeding (*P* = 0.001) or walking (*P* = 0.008) but not grooming (*P* = 0.154).

**Table 3:** Differences in height and radius with respect to fly type (fertile mass-reared, sterile mass-reared, and wild), behavior (resting, grooming, walking, and feeding), and the interaction between these two variables.

Response	Factor	*F* value	DF	*P* value
Height	Fly type	12.46	2, 945.01	4.6 × 10^–6^
	Behavior	10.15	3, 952.48	1.4 × 10^–6^
	Fly type: Behavior	2.09	6, 939.42	0.052
Radius	Fly type	0.24	2, 952.03	0.788
	Behavior	7.26	3, 957.64	8.1 × 10^–5^
	Fly type: Behavior	0.59	6, 945.37	0.739

Significance of these fixed factors was assessed using linear mixed modeling, with both models including day as a random factor. F-statistics were calculated using type III ANOVA with Satterthwaite’s method.

### Leaf surface by abiotic condition and time of day

**Figure 5:**
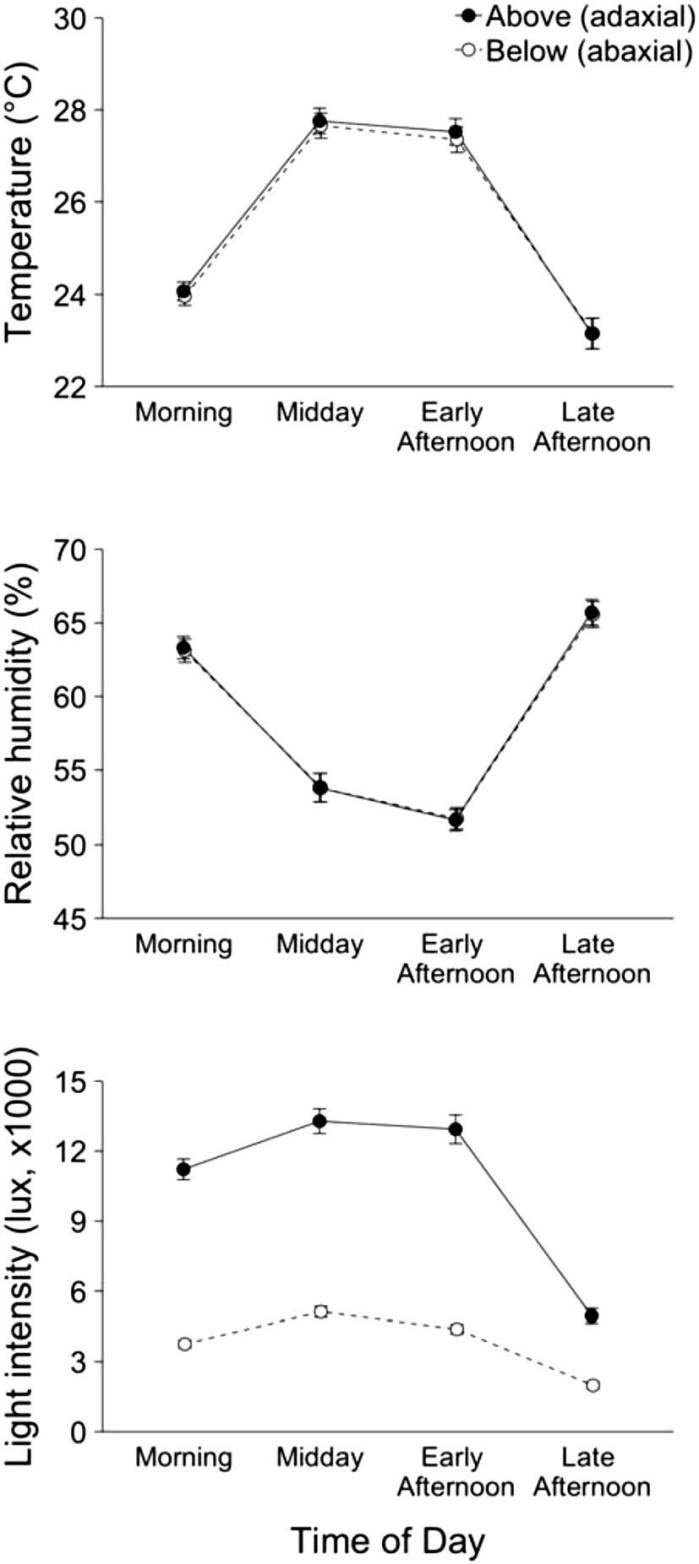
Average (± SE) abiotic conditions (temperature, relative humidity, and light intensity) above (adaxial) and below (abaxial) leaf surfaces where flies were observed throughout the day.

Abiotic conditions changed significantly through the day, and light intensity (but not temperature or humidity) was strongly associated with either upper or lower leaf boundaries (Fig. 5). Temperatures changed through the day (*P* < 0.001), with temperatures in the morning lower than at midday and early afternoon, but higher than in late afternoon. Temperatures at midday and early afternoon were not different (*P* = 0.771). Relative humidity also changed through the day (*P* < 0.001), with humidity in the morning higher than at midday and early afternoon, but lower than in late afternoon. A non-significant trend suggested humidity at midday was higher than at early afternoon (*P* = 0.0572). Light intensity was greater on the upper side of leaves compared to the lower side of leaves (*P* < 0.001). Light intensity changed through the day (*P* < 0.001), with light intensity in the morning lower than at midday and early afternoon, but higher than in late afternoon. Light intensity at midday and early afternoon were not different (*P* = 0.463). A significant interaction between light intensity and time of day revealed that the difference in light intensity between upper and lower leaf surfaces was greater in the morning, midday, and early afternoon compared to the late afternoon (*P* < 0.001).

Flies were observed more often on the lower (abaxial) compared to the upper (adaxial) leaf surfaces during the morning (256/328 = 78.0%, p < 0.001), midday (268/327 = 82.0%, p < 0.001), early afternoon (296/362 = 81.8%, p < 0.001) and late afternoon (313/359 = 87.2%, p < 0.001) sessions across all fly types.

### Leaf surface by behavior

Leaf surface varied significantly by behavior across all fly types, with the upper (adaxial) leaf surface utilized more often for feeding (*P* < 0.001) and the lower (abaxial) leaf surface utilized more often for resting (*P* < 0.001), grooming (*P* < 0.001), and walking (*P* < 0.001) (Table 4).

**Table 4:** Binomial test results of leaf surface (upper = adaxial and lower = abaxial) by fly type and common behaviors.

Behavior	Fly type	N upper	N lower	% lower	Dominant Leaf surface	*P* value
Resting	Fertile	10	208	95.4	Lower	< 2.2 × 10^–16^
	Sterile	14	179	92.7	Lower	< 2.2 × 10^–16^
	Wild	7	326	97.9	Lower	< 2.2 × 10^–16^
Grooming	Fertile	9	91	91.0	Lower	< 2.2 × 10^–16^
	Sterile	8	69	89.6	Lower	3.14 × 10^–13^
	Wild	14	108	88.5	Lower	< 2.2 × 10^–16^
Walking	Fertile	18	43	70.5	Lower	0.0019
	Sterile	16	35	68.6	Lower	0.0110
	Wild	27	31	53.4	**–**	0.6940
Feeding	Fertile	31	12	27.9	Upper	0.0054
	Sterile	21	4	19.0	Upper	0.0009
	Wild	68	27	28.4	Upper	3.11 × 10^–5^

*P* values were calculated based on the null hypothesis of equal probability (*P* = 0.5) that flies occurred on either upper or lower leaf surfaces.

## Discussion

The canopy locations occupied by *Bactrocera tryoni* adults changed systematically through the day and changes in location were correlated with changes in abiotic conditions. Leaves lower in the tree canopy had lower temperature and light intensity and higher relative humidity, and these locations were more frequently occupied by *B. tryoni* when overall ambient conditions were hotter, drier, and brighter. The flies did not modulate their canopy locations horizontally based on distance from the canopy centers, and there was only a weak association between temperature, humidity, and light intensity and distance from the center of the canopies. While abiotic factors were stronger predictors of fly distribution than time of day, substituting time of day produced similar recommended models. The morning and late afternoon times were characterized by low temperature and light intensity, and high humidity, whereas the inverse was associated with a high sun angle during midday and early afternoon. Taken together, our findings suggest that flies moved through the canopy to avoid hydric and/or thermal stress from exposure to high temperature, high light intensity, and low humidity conditions. All fly types responded similarly, although wild flies tended to be found higher in the canopies than either fertile or sterile mass-reared flies, which is consistent with a greater tolerance of hotter, drier and brighter conditions.

The movement of flies to lower canopy regions suggests that they were seeking cooler, more humid and less bright conditions. The temperatures observed in our field cages were frequently higher than the range of 28˚C to 31˚C that *B. tryoni* prefers^48^, and flies may therefore have been under sustained hydric and/or thermal stress. Canopy regions with high light intensities may be avoided as the body temperatures of fruit flies can increase quickly when in direct sunlight, as has been noted for *R. pomonella*^62^. In our study, all fly types were most often observed on the underside of leaf surfaces, which was likely to avoid direct sunlight which may increase body temperatures, increase metabolic rate (thereby depleting nutrient reserves), and accelerate water loss. Observations from other fruit fly species suggest similar behavior, as dense foliage with less penetrating light is preferred by *Zeugodacus cucurbitae*^63^, *Bactrocera cacuminata*^30^, *R. pomonella*^64^, and *C. capitata*^65^. Adult *C. capitata* have been observed to locate higher in canopies when temperatures are low and lower in canopies when temperatures are high^31,66,67^, which resembles the trend we observed in *B. tryoni*.

While *B. tryoni* avoided direct sunlight during the hottest times of the day, they were located higher in the canopy in the morning and late afternoon. The morning follows a long period of cooler temperatures at night and flies may benefit from increased metabolism, induced by exposure to higher temperatures in higher regions of the canopies, so they can become active sooner^68,69^. Flies may also benefit from increased metabolism in the late afternoon when preparing for mating activity which commences close to dusk in *B. tryoni*^70^ and is energetically demanding^71,72^. In *C. capitata*, which mates diurnally, males may occupy leaf surfaces in direct sunlight when calling for females to raise their activity levels in order to more effectively attract prospective mates^73^. A similar strategy of increased exposure to metabolism-accelerating conditions to increase sexual performance could be employed by *B. tryoni* males.

While *B. tryoni* were usually found on the undersides of leaves, we observed flies feeding predominantly on the top sides of leaves. Similar observations have been reported for *R. pomonella*, which have been suggested to reflect foraging on carbohydrates leached from leaf tissues that precipitate onto the top sides of leaves lower in canopies^74^. Other sources of nutrients (e.g., bird droppings, honeydew, leaf exudates^75^) may also fall from higher regions of the canopy and accumulate on the top sides of leaves. Bacteria and fungi on leaf surfaces have been suggested as a source of protein for adult fruit flies^76–78^. Bacterial and fungal colonies typically proliferate in microclimates with higher humidity, moderate temperature, and limited solar radiation^79–81^. These conditions were typical of locations lower in the tree canopies that we measured, and therefore may have contained bacteria and fungi that are typically consumed by *B. tryoni*^82–86^. It is possible that beneficial bacteria and fungi are found predominantly on the top surfaces of leaves, although no studies have described the microbial foods of fruit flies with regards to leaf surface or canopy location. Flies of all types in this study moved lowest in the canopy when feeding from leaf surfaces, which allowed them to remain in the shade while feeding on the top of leaves. While light intensity differed between the tops and bottoms of leaves, we found no differences in temperature or relative humidity between top or bottom leaf boundaries. It is possible that more sensitive measuring devices (e.g., thermocouples, thermistors), which are designed to measure the conditions on actual leaf surfaces and not just the air boundary, would uncover significant differences in conditions between the top and bottom surfaces of leaves^7,9,10^. Future studies may investigate this further to understand the preference of *B. tryoni* for top or bottom leaf surfaces.

The present study highlights differences in the responses to abiotic conditions by mass-reared and wild *B. tryoni* that may affect the outcomes of SIT applications. Compared with the resident wild populations, sterile *B. tryoni* released into arid regions could suffer high mortality from hydric and/or thermal stress if suitable microclimates are scarce. Wild *B. tryoni* have previously been found to survive longer than mass-reared *B. tryoni* under desiccation stress^49^, and this is consistent with the differences in microhabitat preferences observed in the present study. Broader dispersal and survival of wild *B. tryoni* over large areas can be strongly influenced by temperature, rainfall, and relative humidity^87–91^. Also, Dominiak et al.^92^ notes that wild *B. tryoni* survive best in tropical and urban habitats due to greater availability of water and humid microclimates. Further studies comparing wild and sterile *B. tryoni* dispersal in nature would be insightful in light of our findings. In our study, irradiating pupae to induce sterility did not appear to affect the diurnal behaviors of *B. tryoni* as differences between sterile and fertile mass-reared flies were not detected. The behavioral discrepancies we observed between fly types appear to result from adaption to mass-rearing conditions. Furthermore, wild flies in this study were held in laboratory conditions along with mass-reared flies until they were released into field cages as 6-day-old adults, which may have affected their thermal preference in the field. Wild *C. capitata* are known to acclimate quickly to new thermal conditions and diurnal fluctuations^93,94^, but the plasticity of thermal tolerance in mass-reared and wild *B. tryoni* warrants further inquiry.

Improving the tolerance of mass-reared *B. tryoni* to thermal extremes would likely have substantial value to SIT programs. In some of the earliest studies of thermal conditioning in insects, Meats^95,96^ observed that adult *B. tryoni* kept in cooler laboratory conditions became tolerant to cold temperatures over time. Later, Fay and Meats^97,98^ compared the survival of *B. tryoni* adults (fertile and sterile mass-reared, and wild) derived from pupae held at either low or moderate temperatures. In both studies they observed an improvement in the survival of cold-conditioned *B. tryoni* outdoors when daily minimum temperatures were low. These studies show that thermal conditioning may be a useful tool for SIT management of *B. tryoni* when cold-weather conditioning is necessary, but additional studies are needed to test conditioning for high-heat and low-humidity^14^. A more developed understanding of high-heat conditioning is available in *Drosophila melanogaster*, in which adults conditioned for high-heat tolerance have greater survival under both hot and cold conditions^99^. The environmental conditions in SIT rearing facilities are set to maximize production, and so avoid stressful extremes and have low variability, and this appears to influence microclimate preferences of *B. tryoni* under natural conditions. When SIT is to be used in climatically severe regions, modification of rearing conditions to include high temperatures and low humidity may yield more effective results despite expected trade-offs with productivity of mass rearing^100^.

Building on the present investigation of systematic movements of *B. tryoni* in tree canopies through the day, there is a need for greater understanding of movements of wild and mass-reared *B. tryoni* on a landscape scale in orchards and non-host vegetation. Releasing sterile *B. tryoni* into habitats suitable for their survival, while applying alternative pest management options in habitats where sterile flies are unlikely to survive, might lead to better management of pest populations and better allocation of resources. Furthermore, proactively manipulating habitats near cropping areas to improve sterile *B. tryoni* survival may be beneficial. Previous studies on the resting sites of *Z. cucurbitae* have greatly enhanced the management of this species, enabling treatment of resting sites preferred by wild flies with baits or insecticides^63,101,102^. Following similar methodologies, plant species that offer the best microhabitat shelters for sterile *B. tryoni* may be identified and grown as border crops in areas where the management of *B. tryoni* by SIT is desired.

## Supplementary Information

Supplementary Information.
