# Supplementary material for: Canopy distribution and microclimate preferences of sterile and wild Queensland fruit flies
Source: Sci Rep. 2021 Jun 21;11:13010. doi: 10.1038/s41598-021-92218-8 (PMC8217526; doi:10.1038/s41598-021-92218-8)
Supplement: Supplementary file 1 — Supplementary Information. [file 41598_2021_92218_MOESM1_ESM.docx]

**Supplementary materials**

**Table S1** Environmental condition in Auburn, Sydney by the month and year when infested loquats were collected. Temperatures are given as average daily minima or maxima for the month, and precipitation is given as summed total for the month.

| Collection Date | High temperature (Mean °C) | Low temperature (Mean °C) | Precipitation (monthly total mm) |
| --- | --- | --- | --- |
| March/2008 | 26.7 | 16.6 | 46.8 |
| February/2009 | 27.5 | 19.3 | 126.0 |
| January/2017 | 31.6 | 20.9 | 39.8 |
| March/2017 | 26.0 | 19.2 | 363.2 |

**Table S2** To investigate differences between replicates from 2008 (replicates 1-3), 2009 (replicates 4-6), and 2017 (replicates 7-10), including the mass-reared and wild flies used in the replicates that were separated by 8 years, we ran two mixed-model ANOVAs testing 1) height and 2) radius. We included the fixed factors fly type, replicate, and the interaction between fly type and replicate. We found no interaction between fly type and replicate, suggesting there was no difference between the flies sourced in studies from 2008, 2009 and 2017. Potential differences between replicates were also compared using graphical methods (see Figures S1-S2).

| Response | Treatment | *F* value | DF | *P* value |
| --- | --- | --- | --- | --- |
| Height | Fly type | 20.00 | 2, 937 | <0.001 |
|  | Replicate | 11.20 | 1, 937 | <0.001 |
|  | Fly type:Replicate | 0.91 | 1, 937 | 0.569 |
| Radius | Fly type | 1.76 | 2, 937 | 0.172 |
|  | Replicate | 10.19 | 1, 937 | <0.001 |
|  | Fly type:Replicate | 0.90 | 1, 937 | 0.582 |


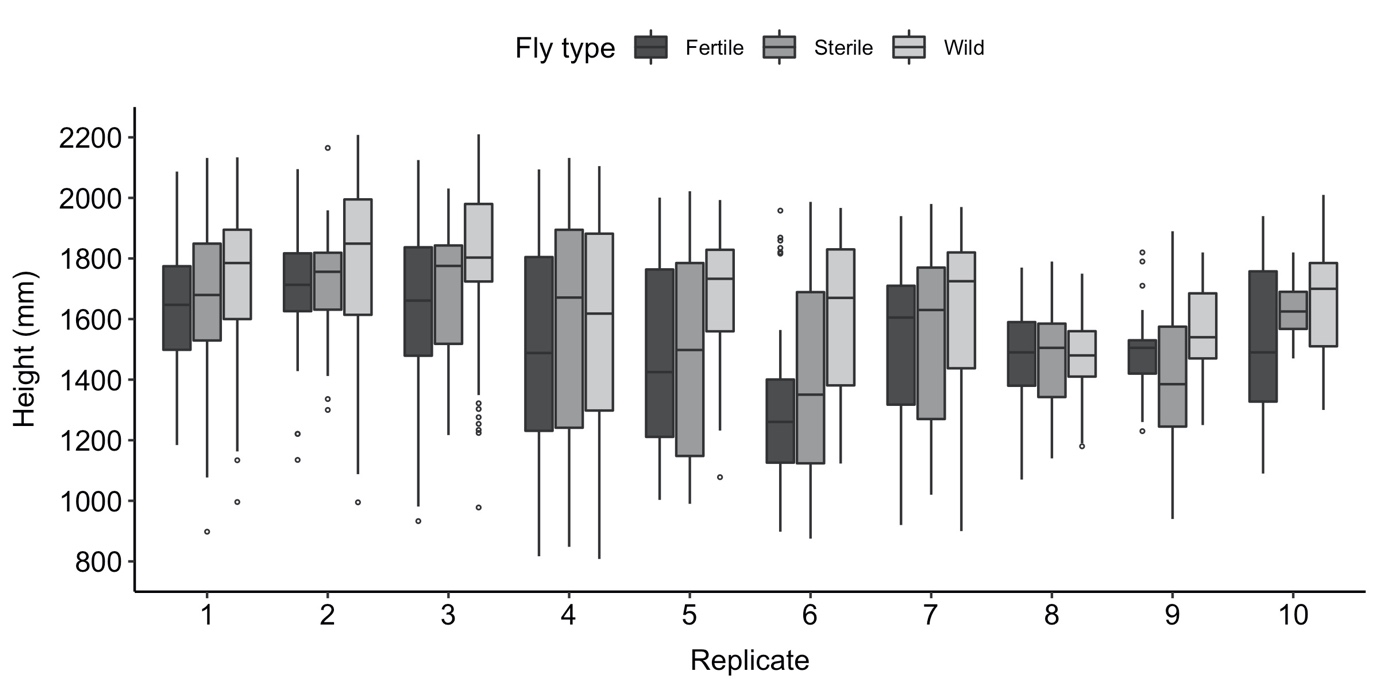


**Fig. S1** Distances from the ground (=height) of observed flies by replicate and fly type.


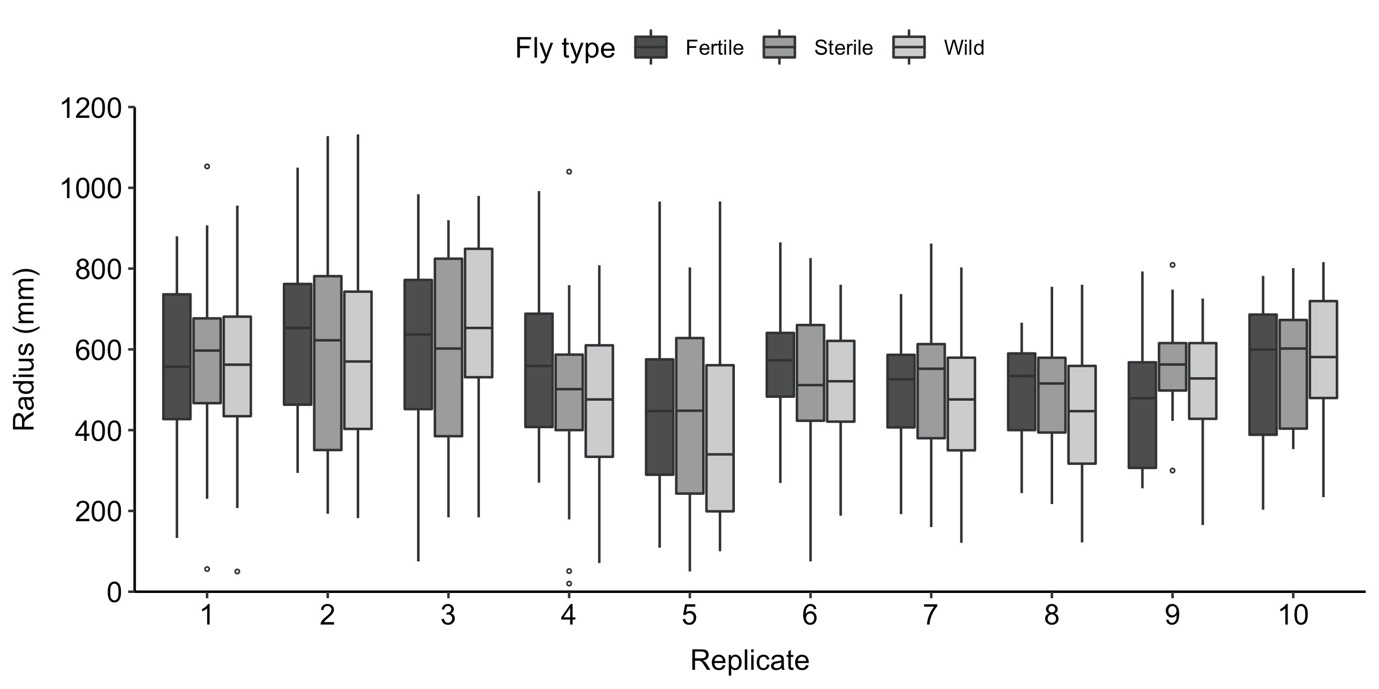


**Fig. S2** Distances from the canopy center (=radius) of observed flies by replicate and fly type.


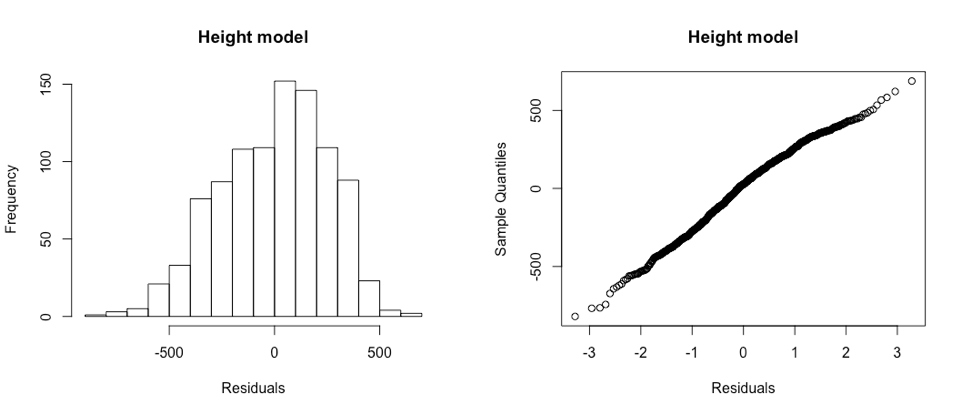


**Fig. S3** Distribution of residuals for final height model.


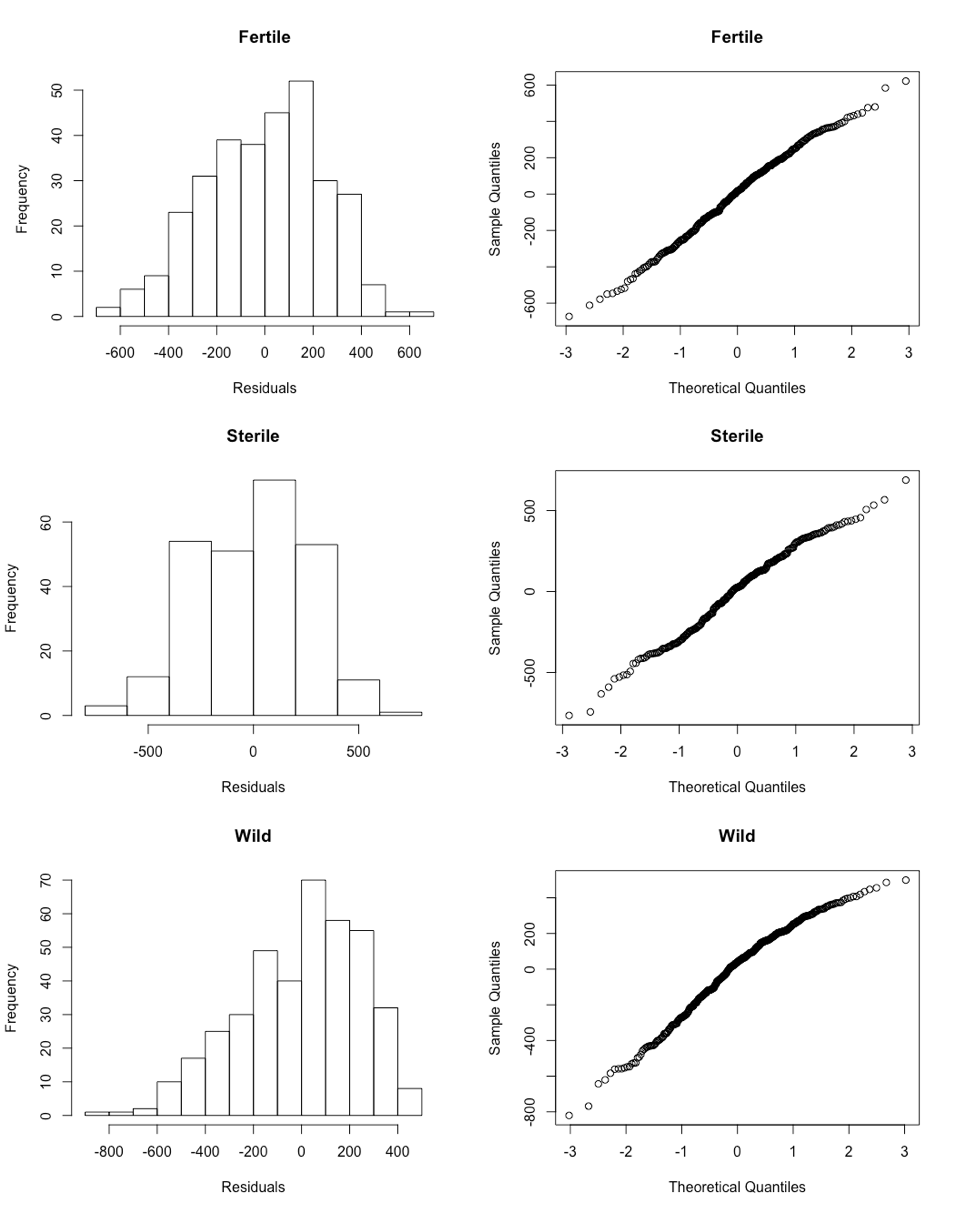


**Fig. S4** Distribution of residuals for final height model separated by fly type.


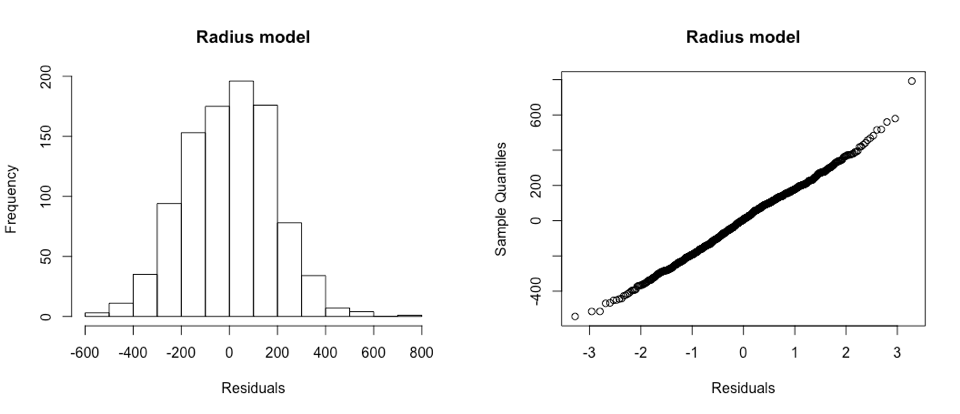


**Fig. S5** Distribution of residuals for final radius model.

**Table S3** Corrected Akaike Information Criterion (AICc) values for fixed factors (age, fly type, sex, temperature, humidity, and light intensity), all 2-way interactions, and random factor ‘day’ by the response variable height. All model permutations (N=40,069) were tested; the 5 models with the lowest AICcΔ are shown here.

| int | a^1^ | f^2^ | s^3^ | t^4^ | h^5^ | l^6^ | a:f | a:s | a:t | a:h | a:l | f:s | f:t | f:h |
| --- | --- | --- | --- | --- | --- | --- | --- | --- | --- | --- | --- | --- | --- | --- |
| 3183 |  | x | x | x | x | x |  |  |  |  |  |  |  |  |
| 3732 |  | x | x | x | x | x |  |  |  |  |  |  |  |  |
| 3222 |  | x | x | x | x | x |  |  |  |  |  |  |  |  |
| 3766 |  | x | x | x | x | x |  |  |  |  |  |  |  |  |
| 3139 |  | x | x | x | x | x |  |  |  |  |  |  |  |  |

| f:l | s:t | s:h | s:l | t:h | t:l | h:l | df | Log(*L*) | AICc | Δ*_i_* | *w_i_* |
| --- | --- | --- | --- | --- | --- | --- | --- | --- | --- | --- | --- |
|  |  |  |  | x | x |  | 11 | -6705.82 | 13433.9 | 0.00 | 0.024 |
|  |  |  |  | x | x | x | 12 | -6704.97 | 13434.3 | 0.36 | 0.020 |
|  | x |  |  | x | x |  | 12 | -6704.99 | 13434.3 | 0.39 | 0.020 |
|  | x |  |  | x | x | x | 13 | -6704.15 | 13434.7 | 0.77 | 0.016 |
|  |  | x |  | x | x |  | 12 | -6705.20 | 13434.7 | 0.81 | 0.016 |

^1^ Age

^2^ Fly type

^3^ Sex

^4^ Temperature

^5^ Humidity

^6^ Light Intensity

**Table S4** Corrected Akaike Information Criterion (AICc) values for fixed factors (age, fly type, sex, and time of day), all 2-way interactions, and random factor ‘day’ by the response variable height. All model permutations (N=113) were tested; the 5 models with the lowest AICcΔ are shown here.

| int | a^1^ | f^2^ | s^3^ | d^4^ | a:f | a:s | a:d | f:s | f:d | s:d | df | Log(*L*) |
| --- | --- | --- | --- | --- | --- | --- | --- | --- | --- | --- | --- | --- |
| 1529 |  | x | x | x |  |  |  |  | x |  | 15 | -6727.88 |
| 1548 |  | x |  | x |  |  |  |  | x |  | 14 | -6729.74 |
| 1514 |  | x | x | x |  |  |  | x | x |  | 17 | -6727.08 |
| 1526 |  | x | x | x |  |  |  |  | x | x | 18 | -6726.29 |
| 1587 | x | x | x | x |  |  |  |  | x |  | 18 | -6726.31 |

| AICc | Δ*_i_* | *w_i_* |
| --- | --- | --- |
| 13486.3 | 0.00 | 0.310 |
| 13487.9 | 1.66 | 0.135 |
| 13488.8 | 2.54 | 0.087 |
| 13489.3 | 3.05 | 0.067 |
| 13489.3 | 3.07 | 0.067 |

^1^ Age

^2^ Fly type

^3^ Sex

^4^ Time of day

**Table S5** Corrected Akaike Information Criterion (AICc) values for fixed factors (age, fly type, sex, temperature, humidity, and light intensity), all 2-way interactions, and random factor ‘day’ by the response variable radius. All model permutations (N=40,069) were tested; the 5 models with the lowest AICcΔ are shown here.

| int | a^1^ | f^2^ | s^3^ | t^4^ | h^5^ | l^6^ | a:f | a:s | a:t | a:h | a:l | f:s | f:t | f:h |
| --- | --- | --- | --- | --- | --- | --- | --- | --- | --- | --- | --- | --- | --- | --- |
| 471.5 |  |  |  |  |  | x |  |  |  |  |  |  |  |  |
| 493.2 |  |  |  | x |  | x |  |  |  |  |  |  |  |  |
| 634.5 |  |  |  | x |  | x |  |  |  |  |  |  |  |  |
| 468.9 |  |  | x |  |  | x |  |  |  |  |  |  |  |  |
| 470.9 |  |  |  |  | x | x |  |  |  |  |  |  |  |  |

| f:l | s:t | s:h | s:l | t:h | t:l | h:l | df | Log(*L*) | AICc | Δ*_i_* | *w_i_* |
| --- | --- | --- | --- | --- | --- | --- | --- | --- | --- | --- | --- |
|  |  |  |  |  |  |  | 4 | -6404.28 | 12816.6 | 0.00 | 0.098 |
|  |  |  |  |  |  |  | 5 | -6403.79 | 12817.6 | 1.02 | 0.059 |
|  |  |  |  |  | x |  | 6 | -6403.10 | 12818.3 | 1.68 | 0.042 |
|  |  |  |  |  |  |  | 5 | -6404.20 | 12818.5 | 1.84 | 0.039 |
|  |  |  |  |  |  |  | 5 | -6404.28 | 12818.6 | 2.02 | 0.036 |

^1^ Age

^2^ Fly type

^3^ Sex

^4^ Temperature

^5^ Humidity

^6^ Light Intensity

**Table S6** Corrected Akaike Information Criterion (AICc) values for fixed factors (age, fly type, sex, and time of day), all 2-way interactions, and random factor ‘day’ by the response variable radius. All model permutations (N=113) were tested; the 5 models with the lowest AICcΔ are shown here.

| int | a^1^ | f^2^ | s^3^ | d^4^ | a:f | a:s | a:d | f:s | f:d | s:d | df | Log(*L*) |
| --- | --- | --- | --- | --- | --- | --- | --- | --- | --- | --- | --- | --- |
| 560.7 |  |  |  |  |  |  |  |  |  |  | 3 | -6407.42 |
| 557.7 |  |  | x |  |  |  |  |  |  |  | 4 | -6407.31 |
| 566.4 |  | x |  |  |  |  |  |  |  |  | 5 | -6407.10 |
| 554.9 |  |  |  | x |  |  |  |  |  |  | 6 | -6406.28 |
| 563.7 |  | x | x |  |  |  |  |  |  |  | 6 | -6407.01 |

| AICc | Δ*_i_* | *w_i_* |
| --- | --- | --- |
| 12820.9 | 0.00 | 0.485 |
| 12822.7 | 1.81 | 0.196 |
| 12824.3 | 3.41 | 0.088 |
| 12824.6 | 3.79 | 0.073 |
| 12826.1 | 5.25 | 0.035 |

^1^ Age

^2^ Fly type

^3^ Sex

^4^ Time of day
